# Supplementary material for: Peptide-Resorcinarene Conjugates Obtained via Click Chemistry: Synthesis and Antimicrobial Activity
Source: Antibiotics (Basel). 2023 Apr 18;12(4):773. doi: 10.3390/antibiotics12040773 (PMC10135297; doi:10.3390/antibiotics12040773)
Supplement: Supplementary file 1 [file antibiotics-12-00773-s001.zip › antibiotics-2329883-supplementary.pdf]

antibiotics

Supplementary material

Peptide-Resorcinarene Conjugates Obtained via Click Chemistry: Synthesis and Antimicrobial Activity

Héctor Manuel Pineda-Castañeda [1], Mauricio Maldonado-Villamil [1], Claudia Marcela Parra-Giraldo [2], Aura Lucía Leal-Castro [1], Ricardo Fierro-Medina [1], Zuly Jenny Rivera-Monroy [1] and Javier Eduardo García-Castañeda [1,*]

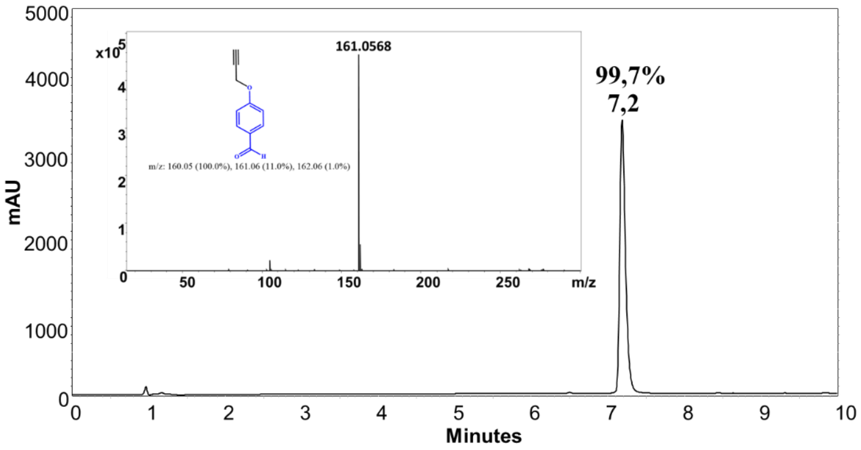

Figure S1. Analysis of p-hydroxyphenyl functionalized with the alkyne group (4-(prop-2-yn-1-yloxy)benzaldehyde), chromatographic profile and mass spectra.

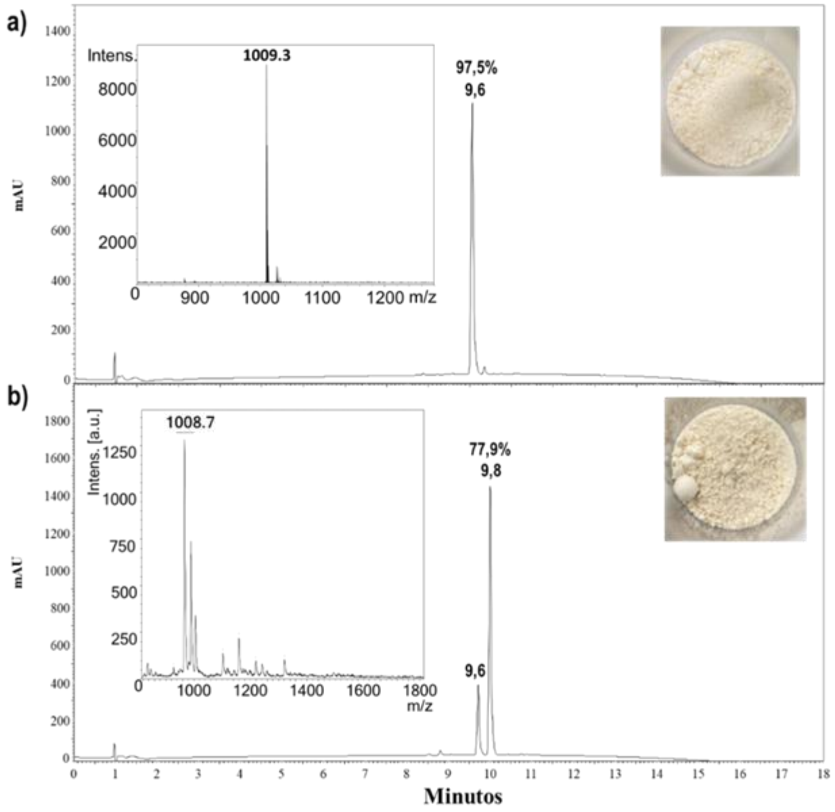

Figure S2. Chromatographic profile and mass spectrum of purified a) *rcct*-chair conformer and b) *rccc*-crown conformer.

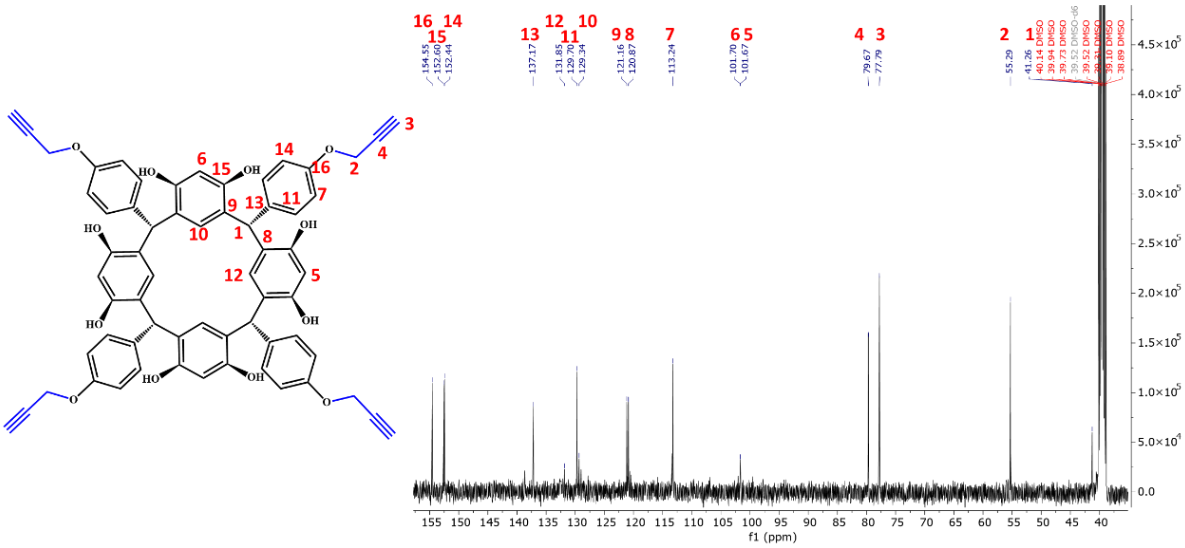

Figure S3. Analysis of C-tetra(p-hydroxyphenyl)calix[4]resorcinarene *rctt* functionalized with the alkyne group, ^{13}C-NMR

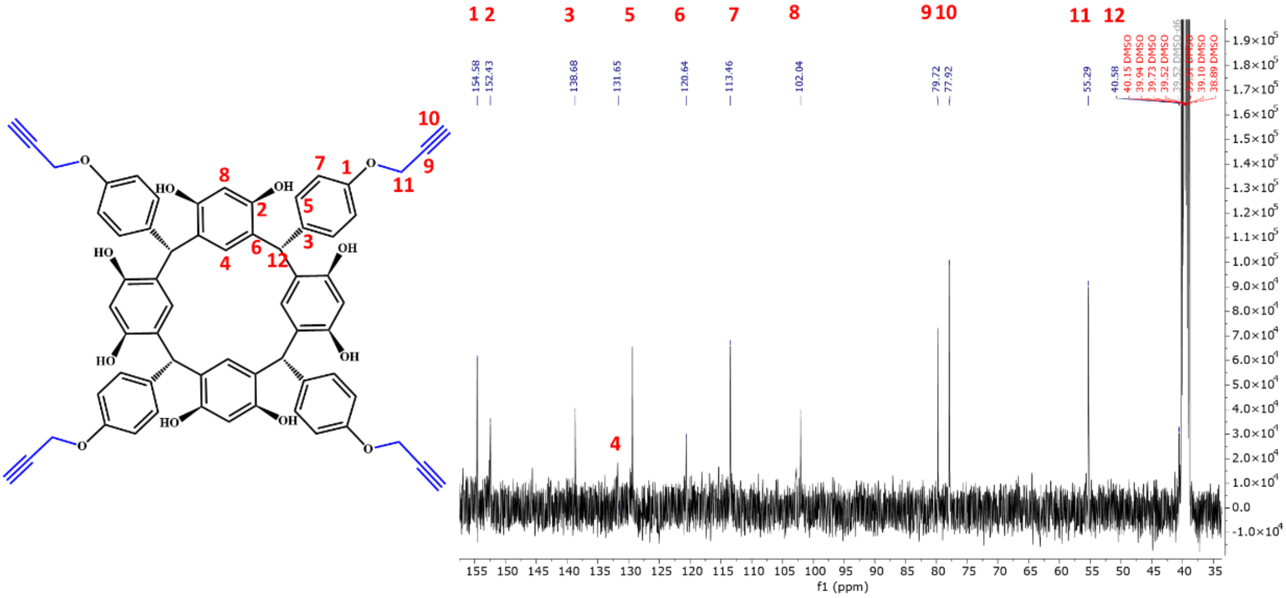

Figure S4. C-tetra(p-hydroxyphenyl)calix[4]resorcinarene *rccc* functionalized with the alkyne group, ^{13}C-NMR.

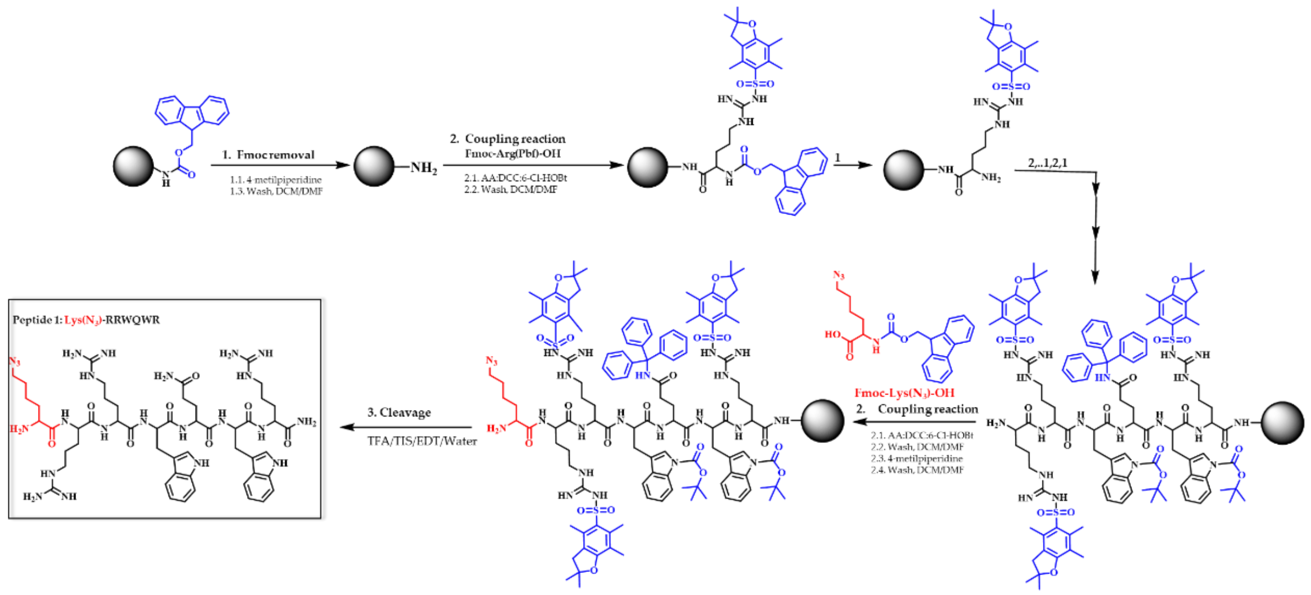

Figure S5. Azide-Peptide synthesis diagram.

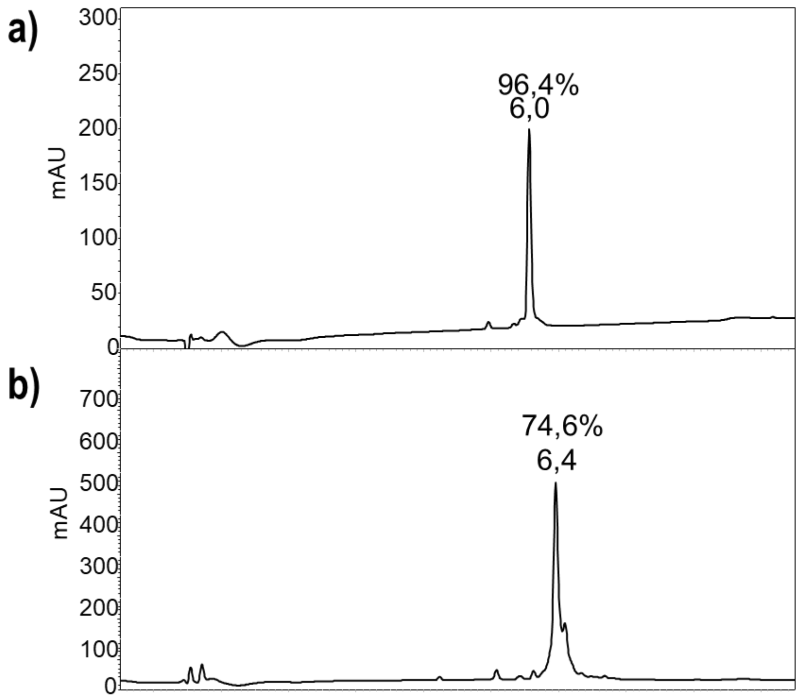

Figure S6. Chromatographic profiles of the peptide-resorcinarene conjugates a) CTpH(F)-(AAC-(K)-RLLR)4 (*rctt*), b) CTpH(F)-(AAC-(K)-RRWQWR)4 (*rctt*)

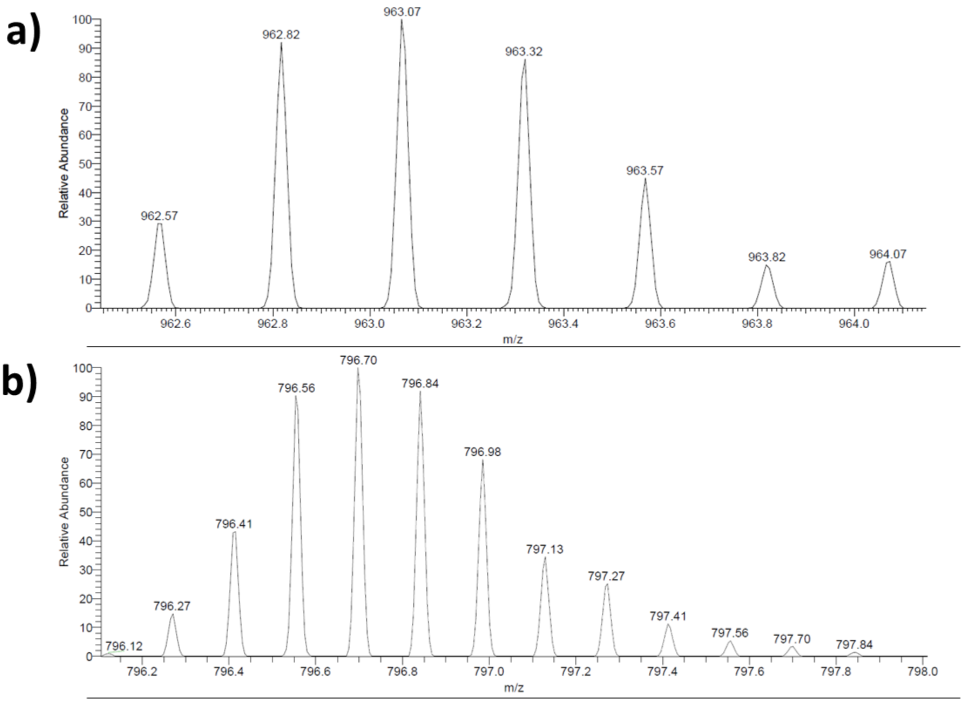

Figure S7. HR-MS of the peptide-resorcinarene conjugates a) CTpH(F)-(AAC-(K)-RLLR)$_4$ (*rctt*), b) CTpH(F)-(AAC-(K)-RRWQWR)$_4$ (*rctt*)
